# Pathways from Depression to Elevated Cardiovascular Risk in Older Adults Through Life’s Essential 8 Model

**DOI:** 10.1093/geroni/igaf122.4384

**Published:** 2025-12-31

**Authors:** Navin Kaushal, Lakshmi Naliyat, Parvati Naliyatthaliyazchayil, Donya Nemati

**Affiliations:** Indiana University, Indianapolis, Indiana, United States; Indiana University-Indianapolis, Indianapolis, Indiana, United States; Indiana University-Indianapolis, Indianapolis, Indiana, United States; Ohio State University, Columbus, Ohio, United States

## Abstract

**Introduction:**

Depression is the leading cause of disability globally and increases the risk for cardiovascular disease, which is the leading cause of mortality. While it’s known that depression negatively affects preventive behaviors, there’s limited research on its direct and indirect impact on lifestyle factors in a comprehensive model. The Life Essential 8 (LE8) model emphasizes four healthy behaviors: physical activity, nutrition, sleep, and not smoking, which influence four biomarkers: LDL cholesterol, BMI, fasting glucose, and systolic blood pressure. Guided by LE8, this study aimed to investigate how depression affects health behaviors and biological factors among older adults.

**Methods:**

The analysis used NHANES data (2021-2023) on community-dwelling older adults in the U.S. (n = 887). A multi-mediation path analysis model was developed where biological factors (LDL cholesterol, BMI and glucose levels) mediate the relationship between systolic blood pressure and health behaviors (nutrition, physical activity, sleep and smoking), predicted by depression assessed by Patient Health Questionnaire-9 (PHQ-9).

**Results:**

Higher scores in depression was found to predict less physical activity (β=-.11, p=.04), worse sleep (β=-.10, p=.04), worse nutrition (β=-.10, p=.04), and greater smoking (β=-.08, p=.04). Accounting for total effects found depression to predict higher BMI (β=.07, p=.022), LDL cholesterol (β=.09, p=.012), and blood glucose levels (β=.11, p=.009).

**Conclusion:**

Depression functioned as barrier to all four preventive health behaviors of the LE8 model and additionally contributed to worsening three biological factors. These findings bring attention to the importance of incorporating intervention components to potentially alleviate depressive symptoms to increase likelihood of successful health behavior adoption.

